# Genetic Diagnosis and Prenatal Diagnosis of a Rare FVIII Family With Haemophilia A

**DOI:** 10.1111/jcmm.70275

**Published:** 2024-12-12

**Authors:** Yaya Yang, Yidan Wang, Jian Gao

**Affiliations:** ^1^ Reproductive Genetics Department Hebei General Hospital Shijiazhuang Hebei China

**Keywords:** factor VIII, genetic diagnosis, Haemophilia A, prenatal diagnosis

## Abstract

It is very difficult to identify the genetic variation of haemophilia A. We examined a case report from a sizable, uncommon haemophilia family, analysing the application of DHPLC in the diagnosis of haemophilia A. The comprehensive clinical data and laboratory assessments of the proband within the family were meticulously compiled. Subsequent to this, tests were conducted to evaluate the activated partial prothrombin time (APTT) and the clotting activity of coagulation factor VIII (F VIII: C). Polymerase chain reaction (PCR) techniques were employed to identify any inversions within the F8 gene's introns 22 and 1. Thereafter, direct sequencing methodology was utilised to sequence all exons of the F8 gene. To analyse the copy number variations across all exons of the F8 gene, a multiple PCR combined with denaturing high‐performance liquid chromatography (DHPLC) approach was adopted. In addition, specific pathogenic mutations predisposing to progenitor cell disorders were screened in family members. The APTT of the proband was 60 s. F VIII: C is < 1%. It was found that the progenitor F8 gene exon 14 had a 226 bp insertion sequence, which was of unknown origin and was a pathogenic mutation. The analysis combined with this family situation is consistent with the expectation. The mutation was used as the detection target to complete the prenatal diagnosis. The pathogenic mutation found in this family is a rare large fragment insertion mutation. It is necessary to combine multiple experimental methods to improve the success rate of genetic diagnosis of haemophilia A.

## 1 Introduction

Haemophilia A (HA), also known as Factor VIII (FVIII) deficiency, is an X‐linked recessive disorder characterised by a deficiency in coagulation FVII. Clinically, individuals with HA exhibit a propensity for prolonged bleeding following injury, tooth extraction or surgery, as well as spontaneous bleeding episodes and delayed healing of first‐degree wounds. The timing of diagnosis and the frequency of bleeding incidents in patients are indicative of the functional level of the clotting factor, and patients are typically categorised as having severe (F VIII: C < 1%), moderate (F VIII: C 1%–5%) or mild (F VIII: C > 5%–25%) HA [1]. What is the most common clinical manifestation of severe hemophilia is spontaneous hemorrhaging from the joints or deep muscle tissue. Typically, patients are identified before reaching the age of 2, and in the absence of prophylactic interventions, they may endure two to five bleeding episodes per month. In the moderate category, spontaneous haemorrhage is infrequent; however, minor traumas can precipitate protracted or delayed bleeding, generally observed before the age of 5 or 6. These patients exhibit a variable frequency of bleeding events, typically ranging from once per month to once per annum. The mild form of the condition is characterised by the absence of spontaneous haemorrhagic symptoms; the bleeding events typically occur during surgical procedures or tooth extraction, when there is no treatment before or after surgery. Diagnosis is commonly made during adulthood or later in life. Patients often experience more bleeding episodes during childhood or adolescence than in adulthood. Approximately 10% of females who are carriers of HA are at risk of bleeding, even if it is mild, making them symptomatic carriers. Following major trauma or invasive surgery, patients are prone to prolonged or excessive bleeding regardless of severity. Due to the lack of a definitive cure for HA, after identifying the pathogenic mutation in the F8 gene, it is crucial to perform prenatal diagnosis on the pregnant women carrying the pathogenic gene mutation. Prenatal diagnosis can effectively prevent the transmission of the pathogenic gene, thereby reducing the birth of affected children, lowering the incidence rate and enhancing the quality of life for HA patients by providing early preventive treatment postnatally, aiming for the possibility of clinical cure.

## 2 Case Report

This family initially sought our hospital's services in December 2018. The pregnant woman with 3 months of intrauterine pregnancy, mainly because there were four haemophilia patients in the fourth generation of the family, who came to our department for genetic counselling and requested prenatal diagnosis. The age distribution of this haemophilia family ranged from 3 years old (IV8) to 36 years old (III5), and one person (I1) had passed away. Their clinical manifestations include spontaneous and mild postsurgical bleeding, with spontaneous bleeding episodes ranging from joint to intra‐abdominal haemorrhages. The severity of these symptoms varies greatly among patients, with the most severe cases resulting in joint deformity.

Relevant education was carried out on the family members. After obtaining full understanding and signing the informed consent letter, 5 mL of peripheral venous blood of the family members was collected, 3 mL of which was used for gene detection and the other 2 mL was tested for activated partial prothrombin time (APTT) and coagulation factor VIII clotting activity (F VIII: C) in our hospital. This study has been reviewed by the Ethics Committee of our hospital.

## 3 Methods

### 3.1 Detection of the F8 Gene Intron Inversion

The sequence of proband (II2) was ligated using T4 DNA ligase following digestion with the restriction endonuclease FbaI, and polymerase chain reaction (PCR) was employed to ascertain the inversion of intron 22 and intron 1 within the F8 gene. The inversion of the intron 22 was investigated via HPLC, and the intron 1 inversion was analysed through agarose gel electrophoresis. The primers were custom‐synthesised by Tianyi Huiyuan Biotechnology Co. Ltd.

### 3.2 Copy Number Analysis of F8 Gene

Multiple PCR was performed on DNA samples from the proband and normal male controls using self‐designed primers, followed by semi‐quantitative analysis of the products using DHPLC. The primers were synthesised by Tianyi Huiyuan Biotechnology Co. Ltd.

### 3.3 Point Mutation Analysis of F8 Gene

Direct sequencing approach was employed to analyse 26 exons and 39 promoter regions of the prover F8 gene. The sequencing primers were independently designed and synthesised by Tianyi Huiyuan Biotechnology Co. Ltd. The identified sequence variants were investigated and assessed for pathogenicity within the ClinVar, LOVD and HADB databases. Upon the discovery of a novel mutation, additional 50 healthy male controls were included to exclude the possibility of a benign genetic polymorphism. Subsequently, an exploration of the SNP database was conducted to ascertain the prevalent rate within the population.

### 3.4 Prenatal Diagnosis

When the proband is identified as harbouring a definitive pathogenic gene mutation, prenatal diagnosis is contingent upon whether the presence of the pathogenic mutation in the pregnant woman. When making a prenatal diagnosis, the gender of the fetus is initially ascertained, subsequent to which the suitable technique is employed to ascertain whether the fetus harbours the same disease. Thereafter, short tandem repeat (STR) genetic locus analysis is conducted to eliminate the possibility of maternal contamination, aforementioned particularly pertinent given the X‐linked recessive inheritance pattern of HA, with the DXS6803 site on the X chromosome being the chosen locus for STR analysis.

## 4 Results

### 4.1 Detection Results of Intron 22 Inversion and Intron 1 Inversion of the F8 Gene of the Proband

**FIGURE 1:**
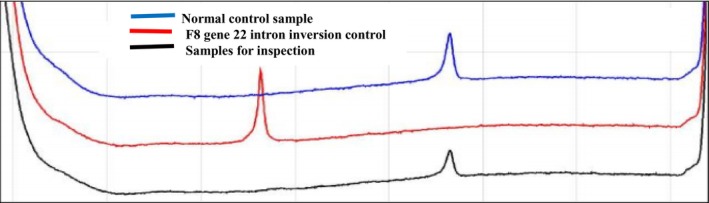
The peak profiles of IS‐PCR products derived from normal male DNA samples, depicted by the blue curve and those derived from 22 inverted male DNA samples, depicted by the red curve. Additionally, the black curve represents the peak profile of IS‐PCR products from DNA samples originating from the proband of the family. Observation of the figure reveals that the detection of the 22 inversion in IS‐PCR products within the family proband aligns with that of normal males. Consequently, it is evident that there is no F8 intron 22 inversion in the proband.

**FIGURE 2:**
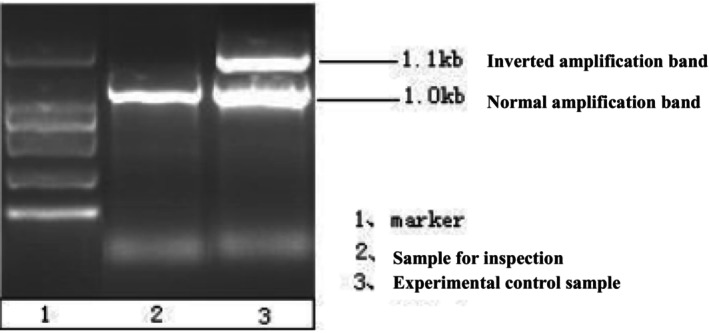
Lane 1 is 1000 bp maker; Lane 2 was the PCR product of DNA samples from the family proband. Lane 3 is the experimental control of mixed positive and negative products. It can be seen from the figure that the fragment sizes of PCR products and negative products in the proband sample are the same, but different from those of positive products. Therefore, there is no F8 intron‐1 inversion in the proband.

No intron 22 inversion and intron 1 inversion were detected in the proband of the family, and the detection results are shown in Figures 1 and 2.

### 4.2 Results of the Large Deletion or Repeated Fragments Detection of the F8 Gene in the Proband

There is no large fragment deletion/duplication was found in each exon of the prover F8 gene.

### 4.3 Detection Results of F8 Gene Point Mutation in the Proband

**FIGURE 3:**
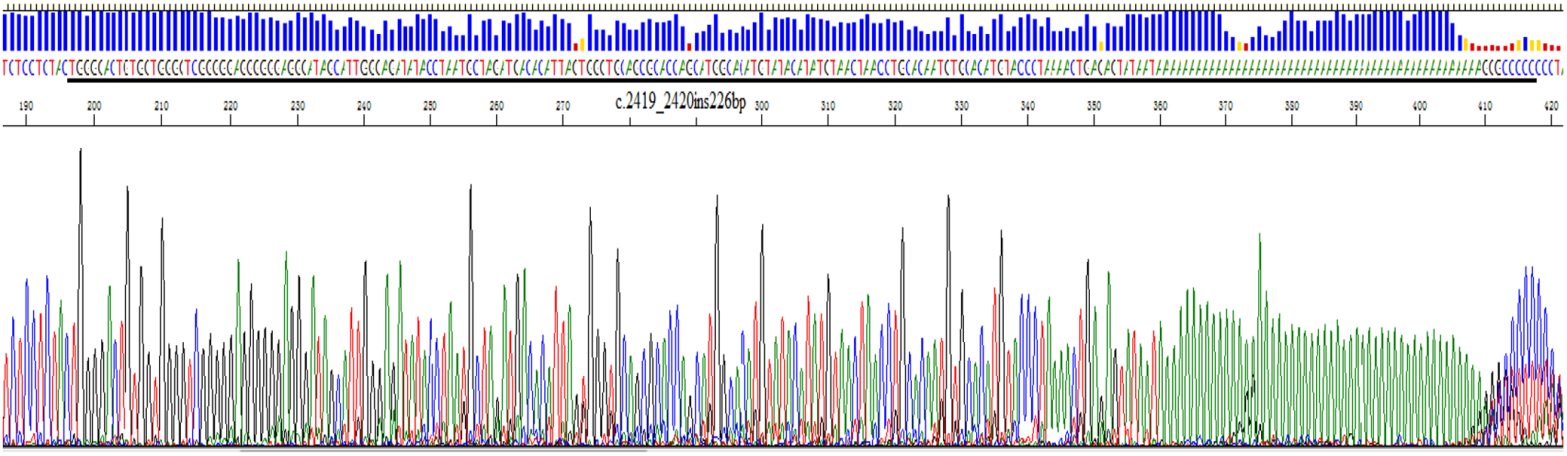
Exon 14 of the F8 gene inserts a large fragment of 226 bp.

An insertion mutation was found in exon 14 of the F8 gene of the proband, the specific site being c.2419_2420ins226bp, the insertion source was unknown (Figure 3). No reports were found in ClinVar database, Lovd database and HADB database, nor in SNP database, and the population carrying rate was less than 0.001%. The same mutation was not found in 50 normal men. According to the analysis of ACMG Classification Standards and Guidelines for Genetic Variants [2], the F8 gene c.2419_2420ins226bp was a pathogenic variant associated with haemophilia A.

**FIGURE 4:**
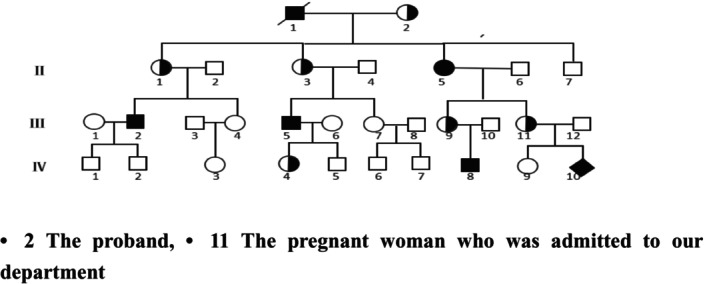
Genogram of the proband—in which III2 is the probator, III11 is the patient who came to our hospital and IV10 is the fetus of this pregnancy.

**FIGURE 5:**
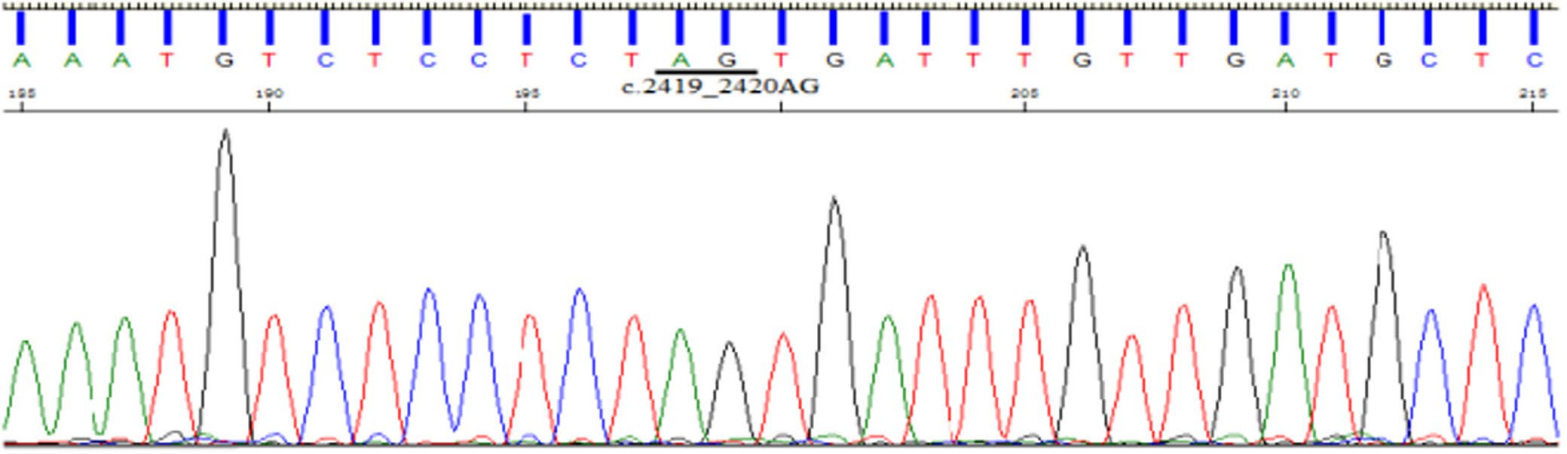
Direct sequencing results of the proband's mother (II1) with no detected insertion mutation.

**FIGURE 6:**
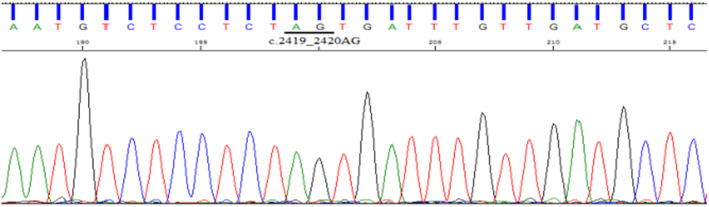
Direct sequencing results of proband family members (II5), with no detected insertion mutation.

**FIGURE 7:**
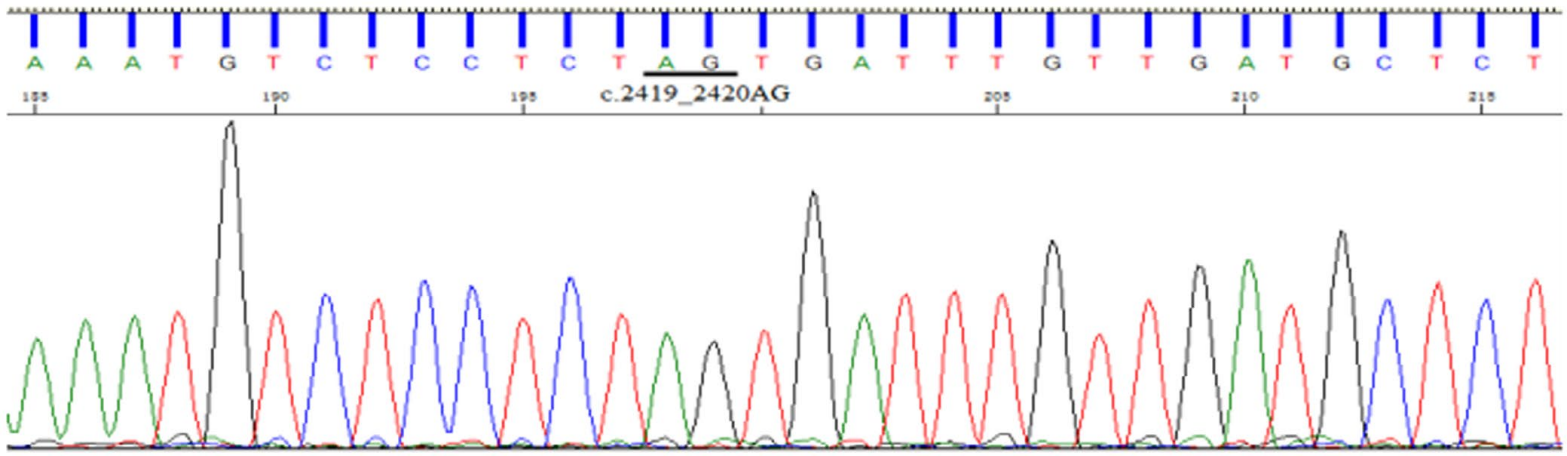
Direct sequencing results of proband family members (II7) with no detected insertion mutation.

**FIGURE 8:**
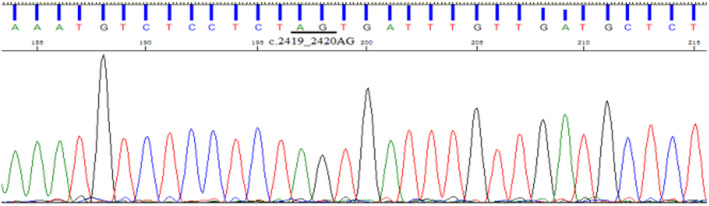
Direct sequencing results of proband family members (III11), with no detected insertion mutation.

The pathogenic mutation sites of the proband's immediate family members were verified: (the family diagram is as follows, Figure 4, the disease status was inferred after consultation). The mother (II1) of the progenitor (III2) did not find the c.2419_2420ins226bp by direct sequencing method. After verification of other family members, there is no c.2419_2420ins226bp was found by direct sequencing in II5, II7 and III11. (Figures 5, 6, 7, 8).

Given the discordance between the verification outcomes and the genetic background of the family, it was determined that the mutation in question did indeed exist. Consequently, specific primers were engineered to target the 226‐bp insertion at the site of the detected mutation, thereby facilitating the amplification of fragments unique to the patient. Additional primers were designed upstream of the F8 gene's c.2419_2420 locus to produce a control product fragment, which served to confirm the presence of the normal F8 gene fragment and the DNA's integrity and sufficiency for the amplification process. The proband served as a positive control, while a normal male individual was utilised as the control for the normal population.

**FIGURE 9:**
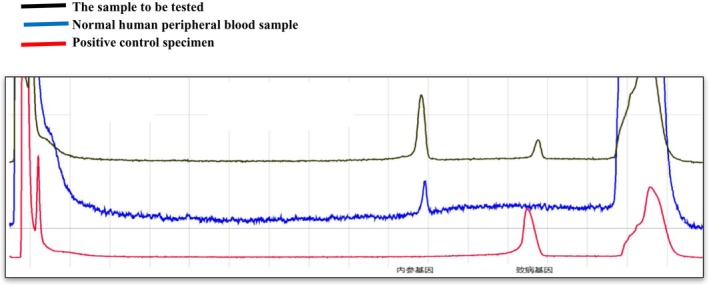
The positive control is represented by the red curve, whereas the negative control is denoted by the blue curve. If the black sample under examination exhibits a peak at the corresponding position of the red curve, it indicates that the sample to be tested can amplify the amplification product of the pathogenic gene, that is, the carrier of the pathogenic gene. Conversely, if the peak is confined solely to the position of the internal reference gene and no peak is observed at the positive control site, it implies that the sample lacks the disease‐causing gene. In the event that peaks do not emerge at either location, the DNA template is considered substandard.

If the sample to be tested has the same pathogenic gene with the proband, the test results are as follows (Figure 9).

**FIGURE 10:**
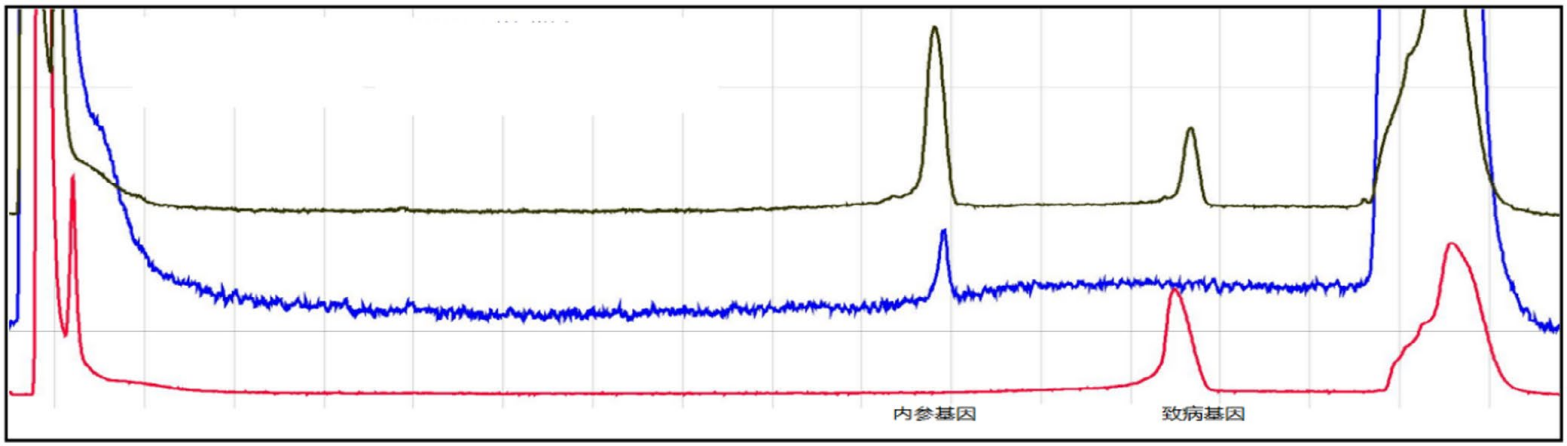
The results of members of family II1. The positive control is the red curve, and the negative control is the blue curve. The sample to be tested has the amplification product peak at the position of the pathogenic gene, that is, the family member carries the same pathogenic gene as the proband.

**FIGURE 11:**
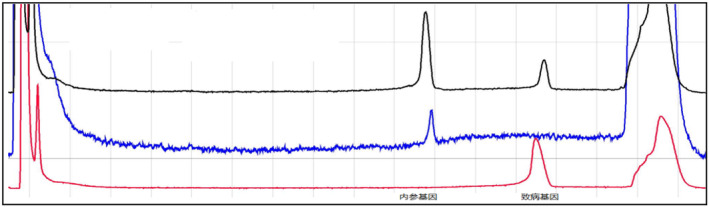
The results of members of family II5. The positive control is the red curve, and the negative control is the blue curve. The sample to be tested has the amplification product peak at the position of the pathogenic gene, that is, the family member carries the same pathogenic gene as the proband.

**FIGURE 12:**
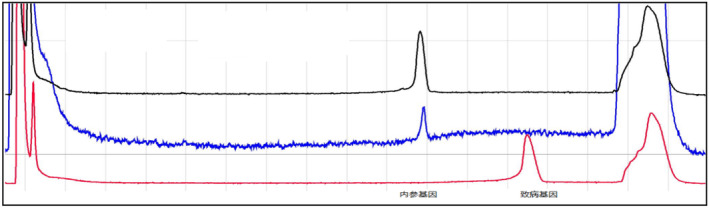
The results of members of family II7. The positive control is the red curve, and the negative control is the blue curve. The sample to be tested only has the amplification product peak at the position of the internal reference gene, that is, the family member does not carry the pathogenic gene of the proband.

**FIGURE 13:**
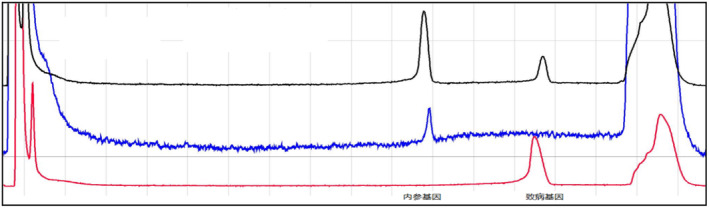
The results of members of family III5. The positive control is the red curve, and the negative control is the blue curve. The sample to be tested has the amplification product peak at the position of the pathogenic gene, that is, the family member carries the same pathogenic gene as the proband.

**FIGURE 14:**
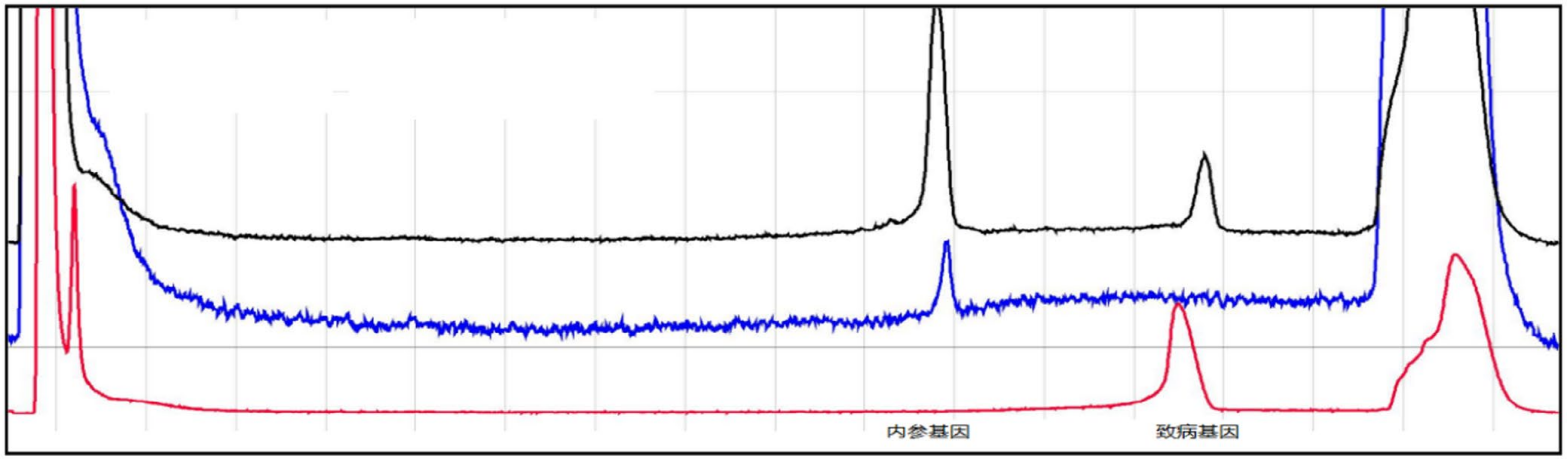
The results of members of family III11. The positive control is the red curve, and the negative control is the blue curve. The sample to be tested has the amplification product peak at the position of the pathogenic gene, that is, the family member carries the same pathogenic gene as the proband.

From Figures 9, 10, 11, 12, 13, 14, the different coloured lines represent the results.

The pathogenic gene test was performed again on the families of the proband, and the results were as follows (Table 1): The verification results of all family members were consistent with the clinical manifestations and the genetic pattern of haemophilia A, and there was co‐segregation between genotype and phenotype, which proved that our detection strategy was effective.

**TABLE 1:** The results of pathogenic gene test on members of the proband's family. The DHPLC results for the family member are as follows (Figures 10, 11, 12, 13, 14).

Family members of the figure	Gender	The clinical presentation/the family history	Results of pathogenic gene detection
II1	Female	The father is HA	Carrying the pathogenic gene
II5	Female	The father is HA	Carrying the pathogenic gene
II7	Male	The father is HA	Not carrying the pathogenic gene
III5	Male	HA	Carrying the pathogenic gene
III11	Female	The mother is HA	Carrying the pathogenic gene

**FIGURE 15:**
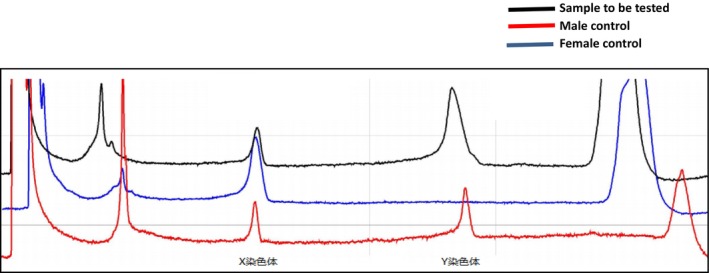
DHPLC results of cord blood samples for sex identification of family member III11. The red curve is the amplification result of normal male, and both X and Y chromosome products were amplified. The blue curve is the amplification results of normal females, with amplification products on the X chromosome but no amplification products on the Y chromosome. The black curve is the cord blood sample of family member III11, and the products of X chromosome and Y chromosome were amplified, which were consistent with the results of normal male. The fetus of family III11 was determined to be male.

**FIGURE 16:**
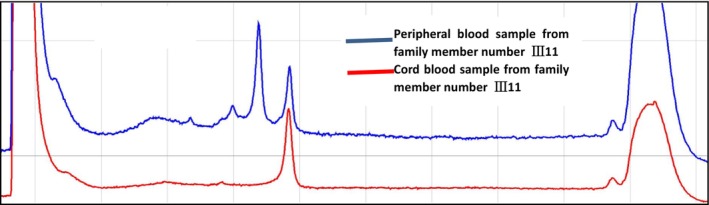
The sequencing results of F8 gene c.2419_2420 locus in the cord blood samples of family member III11. The horizontal line is the insertion part of F8 gene c.2419_2420ins226bp.

**FIGURE 17:**
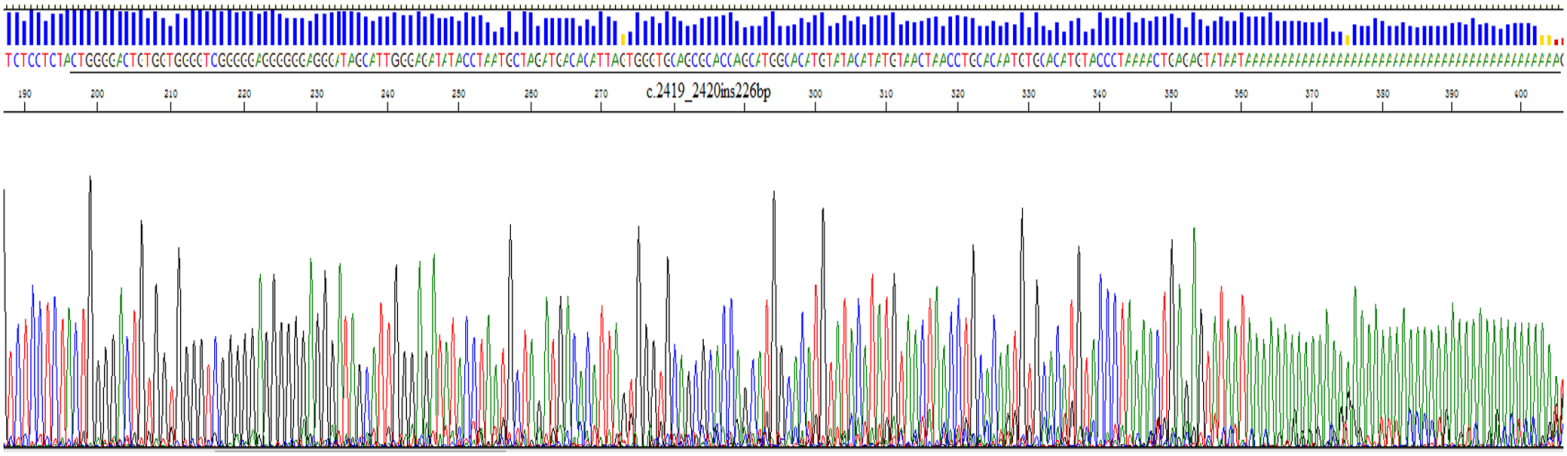
The STR genetic locus analysis of cord blood samples of family member III11. The blue curve is the amplification peak map of peripheral blood of family member III11, and the red is the amplification peak map of cord blood of family member III11. The red curve had only one amplification peak, and only one of the X chromosome amplification products was detected, and the other X chromosome was not amplified, which indicated that there was no maternal DNA mixed in the cord blood sample and maternal contamination could be excluded.

After the proband of family III11 was identified as the carrier of the pathogenic gene, umbilical cord blood was taken at 22 weeks of pregnancy. The obtained umbilical cord blood was subjected to sex identification, pathogenic loci verification and STR genetic locus analysis. After sex determination, the fetus was confirmed to be male (Figure 15). Direct sequencing results suggest the fetus to carry the same insert pathogenic mutation as the proband c. 2419 _2420ins226bp (Figure 16). Analysis of STR genetic loci showed no evidence of maternal contamination in the cord‐blood sample (Figure 17).

## 5 Discussion

Haemophilia A(HA) is A rare X‐linked recessive genetic disease. HA was caused by F8 gene variants clotting Factor VIII (Factor VIII, FVIII) quantity caused by lack of activity and protein function to reduce blood clotting disorder, the disease mainly for male patients [3], the incidence of the HA worldwide is about 1/5000.

F8 gene is located at the end of the long arm of the X chromosome (Xq28), with a total length of 186 kb, consisting of 26 exons, 25 introns and flanking sequences. It is the longest coagulation factor [4], encoding FVII. FVIII is an important cofactor of serine protease factor IX(FIX) in the coagulation process. A tensin complex is formed on the activated platelet membrane, which consists of three A‐type domains, one B‐domain, two C‐type domains and three small acidic a‐type domains in the order A1‐a1‐A2‐B‐a3‐A3‐C1‐C2. Intracellular cleavage produces metal ion‐linked heterodimers composed of heavy (variable length fragments of A1‐A2 and B) and light (A3‐C1‐C2) chains [5]. There are 19 aspartic acid glycosylation sites in the B region [6], and A/C region has a total of six glycosylation‐binding sites. The A and C domains were highly homologous in amino acid sequences among different species. The A domain contains A calcium‐binding site. When the coagulation mechanism in vivo is activated, thrombin activates FVII into FVIIa (activated form of FVII) composed of A1, A2 and A3‐C1‐C2 regions. In the endogenous coagulation pathway, the coagulation factors are activated in sequence after the activation of FXII. The FIXa‐VIIIa‐Ca^2+^‐PL complex is formed [7], which activates coagulation factor X and then initiates a series of coagulation reactions. The C domain has homology with factor V and lectin family, and the C2 domain is the binding site of von Willebrand factor (vWF), phospholipid (PL) and Xa(F10a) [8]. The binding of factor VII complex to vWF can enhance its stability in plasma and prevent its premature degradation. The B domain, which is characterised by a high degree of glycosylation, is encoded by exon 14 (the largest exon in F8), accounting for 43% of the coding region, and contains the thrombin activation site [9]. Tissue factor binds to FVII in the exogenous coagulation pathway to initiate the coagulation pathway and then interacts with other coagulation factors to form prothrombin activator which is to activate the prothrombin, eventually make blood clotting, B domain structure in the process of mature VII F protein will be cut, but the genetic changes that occur in the section still can affect the secretion of F VII protein efficiency and affect blood coagulation function [10].

**FIGURE 18:**
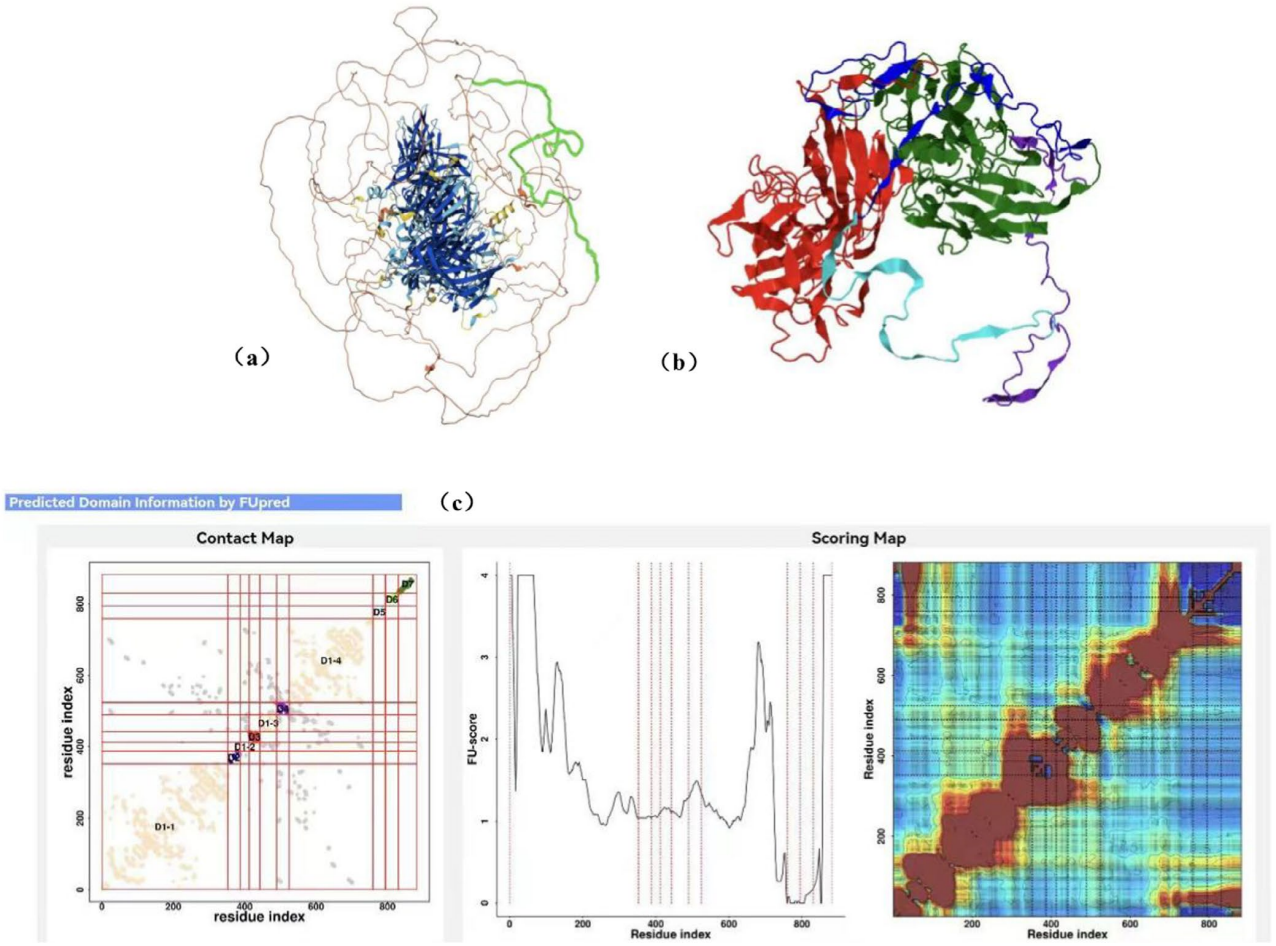
(a) The FVII wild‐type protein was constructed in SWISS‐MODEL. (b) The ultimate full‐length model of large insertion was constructed in I‐TASSER‐MTD. What is predicted the probabilities of inter‐domain interactions between the mutated protein domains 2 and 5 was relatively large, with a predicted value of 0.75. (c). Predicted domain information by FUpred: LOMETS uses FUpred and ThreaDom to predict the domain boundary for the target protein. Where ThreaDom is used for homologous target and FUpred is used for non‐homologous target. The domain prediction result is shown in the predicted contact map (left) and the FUscore for continuous/discontinuous domain are also shown (right).

In this family, a large fragment of 226 bp was inserted in exon 14 c.2419_2420 of the F8 gene, which caused the whole peptide chain to change: This frameshift variant resulted in the introduction of 77 amino acids at position 807, starting from cysteine to isoleucine, which further affected the downstream coding region. The stop codon was changed from 2352 to 883, which caused the premature termination of transcription and translation, resulting in an incomplete and unstable non‐functional polypeptide chain (PVS1). The B domain was destroyed and the light chain A3‐C1‐C2 region was lost, and the length of the protein was significantly shortened (PM4). SWISS‐MODEL is employed to construct a three‐dimensional model of the FVII wild‐type protein, while I‐TASSER MTD is utilised to forecast the ultimate full‐length model of large insertion fragments (Figure 18) and to assess the potential impacts of pertinent pathogenic variants on functional proteins [11, 12].

The variation in clinical gene was not reported in the database, in ExAC database, ClinVar database, Lovd database and HADB database are not found in the general population (PM2), the more highly conserved between species, the site through MutationTast harmfulness forecasting result is 1 (PP3), According to the ACMG guidelines [2], the mutation was identified as pathogenic (PVS1 + PM2 + PM4 + PP3). Of course, there is still a lack of in vitro functional experiments to verify the effect of the mutation on FVII function. At the same time, the clinical severity of patients with the same mutation in this family may be different due to the different periods of FVII factor replacement therapy. The earlier the start of treatment, the milder the phenotype and the longer the treatment time (the higher the risk of inhibitors and the more prone to bleeding) may also be involved in the expression of the severity of the phenotype.

The complexity of HA gene diagnosis is due to the complexity of F8 variants (variable expression and allelic heterogeneity, etc.). Due to the limitation of detection technology and the complexity and heterogeneity of gene mutations, the accurate diagnosis of haemophilia is difficult to reach 100%. At present, the detection methods of HA include direct diagnosis (PCR amplification and screening, etc.) and indirect polymorphic site linkage analysis (using closely linked polymorphic fragments within or outside the FVIII gene, polymorphic markers at mutation sites and the rule of transmission to offspring) to detect proband, carriers and prenatal diagnosis. The relationship between the type of genetic variant and the severity of the phenotype also affects the detection strategy of HA. The most common mutation in the most severe HA (F VIII: C < 1%) was intron22 inversion (Inv22), accounting for 45%–50%, followed by intron 1inversion (Inv1), accounting for about 1%–5%. The majority of severe haemophilia A is caused by intron 22 and 1 inversion, large fragment deletion and nonsense mutation, which often cause the majority of FVII protein deletion and dysfunction. The most common type of mild/moderate haemophilia is missense mutation, small deletion or insertion [13]. Large fragment insertion is rare in the F8 gene mutation database [14], so the detection strategy of HA disease is to first detect intron 22 and 1 inversion. If the result is negative, the point mutation and minimal deletion or duplication of F8 gene are screened. Polymerase chain reaction (PCR) combined with Sanger sequencing was used to verify and screen the mutations in important regions, and finally to detect the presence of large deletions or duplications. In this study, the family of detection strategy is: The FVIII: C activity of the proband (III2) suggested the presence of severe HA. We first detected the inversion of common pathogenic loci of F8 in the proband (III2). The copy number variation of III2 with negative inversion was detected and all exons of F8 gene were sequenced by direct sequencing to identify the pathogenic site of the proband. While the pathogenic variant was not found in the mother (II1) of proband (III2) by direct sequencing, which was verified by other family members, and was not found in II5, II7 and III11 by direct sequencing. However, according to the detection of III1 and the genetic history of the family, we believed that III2 was a male patient. In the process of carrier verification, due to two different DNA template strands, the abnormal template was 226 bp more than the normal one. Under the same conditions, the abnormal template of the female carrier could not compete with the normal template, so it could not be amplified. Therefore, we designed primers for specific amplification of the insertion fragment. Linkage analysis and PCR‐denaturing high‐performance liquid chromatography (DHPLC) were used to verify the pathogenic mutation in the family members and normal controls were also included. DHPLC technology is based on the retention time of heterozygous and homozygous diploid in the column under partial denaturation conditions, to find DNA mutations (heteroduplex DNA and homologous duplex DNA have different melting characteristics. Under partial denaturation conditions, heteroduplex DNA is more volatile due to the presence of mismatch regions, so the retention time in the column is shorter than that of homologous duplex DNA. It is eluted first, showing a bimodal or multi‐peak elution curve in the chromatograph). DHPLC results suggested that the family was in accordance with the genetic model of HA. Prenatal diagnosis should also be performed for the pregnant woman carrying the pathogenic variant (III11): The umbilical cord blood (excluding maternal contamination) was used to determine the sex and mutation type of the fetus by DHPLC and direct sequencing. The results showed that the fetus of the mother carried the same pathogenic mutation, which enriched the mutation spectrum of the F8 gene. At the same time, our study suggests the value of combined application of multiple detection techniques in gene diagnosis, carrier screening and prenatal diagnosis of the HA.

At present, the treatment of HA can be divided into two directions, one is clinical replacement treatment and preventive treatment for existing HA patients and the other is carrier screening and prenatal diagnosis for HA family members. The corresponding current treatment difficulties are the occurrence of inhibitors in the process of replacement therapy: The risk of inhibitor development is associated with different gene domains and different mutation types [15] (the risk of inhibitor development is higher when the FVII protein mutation occurs in the C1 and C2 domains of the light chain region [16], and it is lower when the small insertion or deletion mutation in the polynucleotide sequence of the B domain [17]. Related gene therapy studies have found that deletion of the B domain reduces the risk of FVIII inhibitors; Null mutations—large deletions and nonsense mutations have a higher risk of producing inhibitors, small deletions and insertions have a lower risk), it has been found that the secretion efficiency of FVII with B‐domain deletion is higher than that of the full‐length sequence. Moreover, FVII containing several glycosylation sites in the B domain was more secreted than that without the B domain [18]. FVIII‐Padua is a 23.4 kb tandem repeat mutation in F8, which is associated with high expression of F8 gene and thrombosis, and is a new gene therapy target [19]. Therefore, it has a high application prospect to enhance the expression of FVII protein while losing the B domain gene therapy, and the preventive treatment of haemophilia patients can be truly realised after successful immune tolerance therapy [20]. The second major difficulty in HA is the genetic diagnosis of the proband or the lack of screening of the proband's family carriers, which makes it difficult to carry out the prenatal diagnosis of haemophilia and the eugenics of the potential risk population.

## 6 Conclusion

The pathogenic variation of F8 gene is complex. It is crucial for clinicians to correctly understand HA and to diagnose the disease with a variety of detection techniques for the treatment and prevention of HA.

## Author Contributions

**Yaya Yang:** conceptualization (equal), investigation (equal), methodology (equal), writing – original draft (equal). **Yidan Wang:** data curation (equal), investigation (equal), supervision (equal). **Jian Gao:** conceptualization (equal), funding acquisition (equal), resources (equal), supervision (equal), writing – review and editing (equal).

## Consent

The patient had given informed consent, and the study had been reviewed by an ethics committee.

## Conflicts of Interest

The authors declare no conflicts of interest.

## Data Availability

Data sharing not applicable to this article as no datasets were generated or analysed during the current study.
